# Design of AI System for National Fitness Sports Competition Action Based on Association Rules Algorithm

**DOI:** 10.1155/2022/1375009

**Published:** 2022-09-16

**Authors:** Jianmin Xiang, Litao Tong, Shengfa Zhou

**Affiliations:** School of Physical Education, Shangrao Normal University, Shangrao, Jiangxi 334000, China

## Abstract

In information system construction, online data migration is a very important link. At present, in different fields, people provide protection for online data migration through the way of project management to ensure the speed and efficiency of online migration. However, some problems may occur in the process of online data migration. In the development of contemporary sports, competitive sports, as the high-end stage of sports development, are constantly pursued by ordinary sports enthusiasts. Therefore, in the national fitness activities, how to combine the national fitness and competitive sports data to provide a more professional storage platform is a focus of research but also a problem to be solved in the process of online data migration. Because the data mining ID3 algorithm only supports querying and retrieving RowKey indexes, it does not support non-RowKey column indexing. Therefore, if you want to query non-RowKey indexes, the data mining ID3 algorithm will search the form in the overall scan, but the performance of this method is low. In order to improve the query speed of non-RowKey columns, this paper designs a secondary index function based on HBase. The sports competition action system can retrieve data from the secondary index of the query state, to avoid scanning the whole world and improve the search speed. In this paper, ID3 algorithm is used to combine national fitness and competitive sports data, which provides a guarantee for the migration of competitive sports data in the national fitness system.

## 1. Introduction

With the rapid development of modern information technology, massive amounts of data have been accumulated in many fields. The desire to find the knowledge and laws in the data foundation promotes the development of data mining disciplines and the progress of data mining technology [1]. On this basis, this paper seeks data mapping to solve the online data migration problem. Secondly, this paper proposes a tree evaluation algorithm [2]. The most common algorithm used to classify learning trees is the data mining ID3 algorithm, which uses a top-down division and coverage strategy and uses data retrieval criteria to select individual attributes to ensure that a tree is built [3]. The algorithm is simple and efficient, and the knowledge learned is easy for people to understand. However, when extracting knowledge from a large amount of acquired data, there is a problem of overfitting [4]. Combined with the in-depth analysis of the data mining ID3 algorithm, an improved genetic algorithm-based data mining ID3 algorithm is proposed, and the algorithm is used to model data with network content [5]. The algorithm first uses known genetic rules as genetic means, then uses genetic algorithms to split the existing rule set, then uses the split rule set to create a set of decision trees, and then uses tree group decisions to provide prediction results [6]. Experimental results show that the algorithm has better classification results and can be compared with the results provided by the data mining ID3 algorithm. Finally, this paper designs a storage management platform for the national fitness and sports competition action system. Analyze the necessary and non-functional system requirements for national fitness and then analyze the main use cases of the system based on the use cases [7]. Functional system analysis mainly focuses on the roles of regular system users, fitness coaches, and venue managers. For the analysis of the above requirements, this article provides a comprehensive overview of the system architecture, business architecture, security architecture, and storage architecture [8]. First introduced the overall architecture design of the national fitness system and then introduced the four business functions of designated courses, designated areas, course management, and on-site management [9]. After analyzing the user's malicious operation scenarios and designing system security for these solutions, in order to respond to the company's requirements, the company is included in the business data model. Secondly, the system chooses Spring Cloud as the landing framework of the fitness system microservice architecture. Based on the cloud framework, the business code development of the user module, location module, and course module was performed [10]. Finally, the functions of venue reservation, course reservation, course release, and venue release were individually tested to ensure the business reliability of the national fitness and sports competition action system.

## 2. Related Works

The literature introduces the possibility of creating a comprehensive tracking system for all medical departments and integrating intelligent auxiliary diagnosis into the tracking system [11]. The theoretical and practical analysis shows that the system is feasible and has great development prospects. The system has obtained many suggestions in practice, which point out the direction for future research and development [12]. The literature introduces related methods to compare the new and old similarities of structural models based on the imperfect establishment of the mapping relationship between documents and the insufficient information of the documents themselves, in order to seek data mapping to solve the migration problem and provide examples for reference [13]. The literature introduces the use of open-source and mature ETL Kettle tools for static table data migration and the use of dual-run verification and report verification techniques to verify the quality of data migration. The literature introduces a complete general scheme of exercise-assisted training. This article describes the general system of exercise-assisted training in four modules: domain knowledge, trainee, exercise analysis, and controller [14]. From the perspective of interactive training and series of decision-making, it illustrates how to model the qualification level of subjects in two common yes/no feedback training situations. The principle of calculating the ideal feedback loop and the importance of feedback guidance are put forward so that the trainee can use the most effective training method to solve the expected goal of each skill index below the current skill level [15]. Some research introduces a table tennis auxiliary training system based on a general structure. After participating in the assigned sample experiment, subjects of different levels are recruited, and the level distribution of subjects is calculated according to the actual situation according to the quality of the balls returned by the system and the standard of action. The availability of the platform and the effectiveness of the exercise evaluation method have been initially verified.

## 3. Online Data Migration Model and Data Mining Algorithm Model Design

### 3.1. Online Data Migration Model

This evaluation model uses the system data importance model and divides its importance into two parts. The data file itself has many attributes. These characteristics depend on I/O. These characteristics depend on I/O and assign importance to the data itself in the parameters. The system monitoring process monitor will update these parameters in real time so that the value of the data set *x* can be defined as follows:

(1) $$\begin{array}{c} AV\left(x\right)=\frac{1}{s}+F+T+E\text{.} \end{array}$$

Parameter design: we studied the importance of access frequency F, where F is a variable representing the change order of data access volume in a period of time. For example, *T*_*i*_ represents a certain fixed time period, and the corresponding frequency *f*_*i*_ of data access; *f*_*i*_ can be positive, which means that the amount of data access increases or is negative, which means that the frequency of partial access to the data decreases. Then, in a migration cycle, divide it into *N* parts and define the amount of visits to data set *X* as follows:

(2) $$\begin{array}{c} F=\overset{N}{\underset{i=1}{\sum }}f_{i}=f_{1}+f_{2}+Lf_{n}. \end{array}$$

Time importance *T* can be expressed as follows:

(3) $$\begin{array}{c} T=\overset{n}{\underset{i=1}{\sum }}\frac{1}{T_{i}}=\frac{1}{T_{1}}+\frac{1}{T_{2}}+L+\frac{1}{T_{n}}. \end{array}$$

The total visits *E* represents the number of visits to the statistical data *X* by all users.

In short, the total value of access parameter *E* in *X* data can be expressed as follows:

(4) $$\begin{array}{c} E=\underset{u_{i}\in UX}{\sum }\text{counter}\left(u_{i}\right)\text{.} \end{array}$$

*C* is the number of potential users who can access the data, and *R* (*u*) is the importance of each potential user. The potential importance is directly proportional to the importance of the data set. Therefore, the value of PV data can be defined as follows:

(5) $$\begin{array}{c} PV=\lambda C\left(x\right)+\left(1-\lambda \right)\underset{u\in U}{\sum }R\left(u\right)\text{.} \end{array}$$

Specific analysis: According to the recommended user-based algorithm, users with highly similar thinking may have similar points of interest and browsing habits. That is to say, if a visitor visits the data set *x*, some users have greater similarities with the user of *y*; then these users may also be interested in the data set *x*, that is, they can access the data set *x*.

In this article, let *α*_*vi*_ be the access identification parameter. *g*(*v*, *k*) = ∑_*i*=1_^*n*^*a*_*v*_*i*__*a*_*k*_*i*__ represents the intersection of the data accessed by users *v* and *k*, that is, the total amount of data accessed by the same user. The similarity between k and *k* can be expressed as follows:

(6) $$\begin{array}{c} \text{Similarity}\left(v,k\right)=\frac{g\left(v,k\right)}{h\left(v\right)+h\left(k\right)}\text{.} \end{array}$$

The detailed calculation steps are as follows:

Step 1. If we divide all users into two user groups according to a certain date we visited, *K* represents users who access data *i*, and *V* represents users who do not access data *i*, so the first group of users can be initialized to $\begin{array}{c} K=\left\{k_{1},k_{2},L,k_{n}\right\}\\ V=\left\{v_{1},v_{2},L,v_{m}\right\} \end{array}$. *n* and *m* represent the total number of users in the set.
Step 2. After the user set split is completed, the *K* and *V* sets are crossed according to formula (6) for calculation.
  (7) $$\begin{array}{c} Similar\left(K_{i},V_{j}\right)=\left[\begin{array}{cccc} S_{11} & S_{12} & L & S_{1m}\\ S_{21} & S_{22} & L & S_{2m}\\ S_{n1} & S_{n2} & L & S_{nm} \end{array}\right]\text{.} \end{array}$$
Step 3. Take the user equivalent time (7), use *ξ* (default value 1/2, self-adjustment) as the standard for selecting the equivalent, compare and select the user-defined *u*_*i*_′ whose equivalent is greater than *ξ*, and traverse the matrix and sets:
  (8) $$\begin{array}{c} u_{i}^{\prime }=getUser\left(Similar\left(K_{i},V_{j}\right)\right)=\left\{u_{1},u_{2}\cdots u_{n}\right\}\text{.} \end{array}$$
Step 4. Each user of the system has its own same user set, for example, *u*_1_′={*u*_1_, *u*_2_, *u*_3_}, *u*_3_′={*u*_3_, *u*_4_, *u*_5_}; this set contains repeated internal users. It is the same user as *u*_3_, and duplicate users should not be considered in practice. Therefore, these same users must be removed, and the same user set *U* can be restored through the deduplication operation. Then you can calculate the number of *U* and the same user *C*:
  (9) $$\begin{array}{c} U=distinct\left(u_{1}\cup u_{2}\cup u_{n}^{\prime }\right)\text{,}\\ C=count\left(U\right)\text{.} \end{array}$$

In the PageRank algorithm, the more important the typed page, the more important the current page. In other words, the importance of the linked page is directly proportional to the importance of the current page. Therefore, if this idea is equally applied to evaluate the importance of users, when calculating the PV value, the first step is to calculate the relationship between users. And the more important the same user is, the more popular and important the data they belong to:

(10) $$\begin{array}{c} R\left(u\right)=\sum_{y\in I_{a}}\frac{R\left(y\right)}{\left|O_{y}\right|}\text{.} \end{array}$$

The potential value (*PV*) can be expressed as follows:

(11) $$\begin{array}{c} PV=\lambda C\left(x\right)+\left(1-\lambda \right)\sum_{u\in U}R\left(u\right)\text{.} \end{array}$$

The effective *AV* and *PV* of the data itself determine the meaning of the *DS* data at the same time as follows:

(12) $$\begin{array}{c} DS=\delta AV\left(x\right)+\left(1-\delta \right)PV\text{,} \end{array}$$

where *δ* is the adjusted weighting weight, and its value is 0 ≤ *δ* ≤ 1.

In order for the system to operate normally, sufficient storage space must be provided, namely:

Storage capacity limit:

(13) $$\begin{array}{c} \overset{m}{\underset{i=1}{\sum }}S_{i}<W\text{,} \end{array}$$

where *S*_*i*_ is the total size of the data set by the system and *W* is the total capacity of the system.

**Figure 1:**
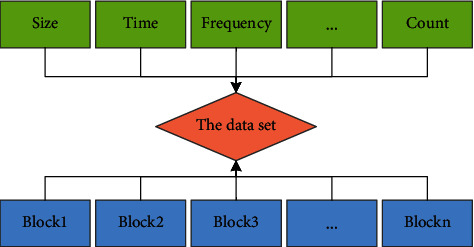
Data set block mapping relationship.

To establish a hierarchical storage architecture, the principle of data migration is particularly important. Before migrating data, a reasonable number of classification additions must be performed. This article uses a file-level method for data mapping. In order to ensure the complete mapping of data, the basic migration unit is also a file, that is, a complete data set contains all data blocks and simultaneously recognizes and maps multiple data. As shown in Figure 1, each data set contains several data blocks, and a mapping relationship is constructed with several attributes at the same time.

**Figure 2:**
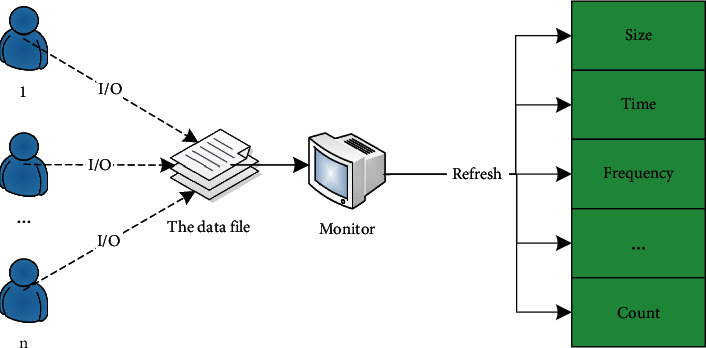
Data file attribute update diagram.

As shown in Figure 1, a set of data files contains multiple data blocks. The size of the data set is based on the value of the number of multiple data blocks. At the same time, as shown in Figure 2, the monitor monitors the I/O used for each block in the data set or updates the data file attribute parameters in real time according to the total number of accesses, the number of users, and the access frequency, thereby updating the ranking of data importance.

System data is affected by data migration and data flow between different environments, which will cause the physical address of the migrated data to change, resulting in a decrease in access rate, and even user access or even data loss. I/O requests can be realized which represents an important indicator of algorithm performance. The higher the access rate, the better the user experience the system can provide. This experiment focuses on testing the data access I/O rate; here, we specify I/O for each user. If the data cannot be located, application O will be lost. If the data can be found accurately, it is called a hit.

**Figure 3:**
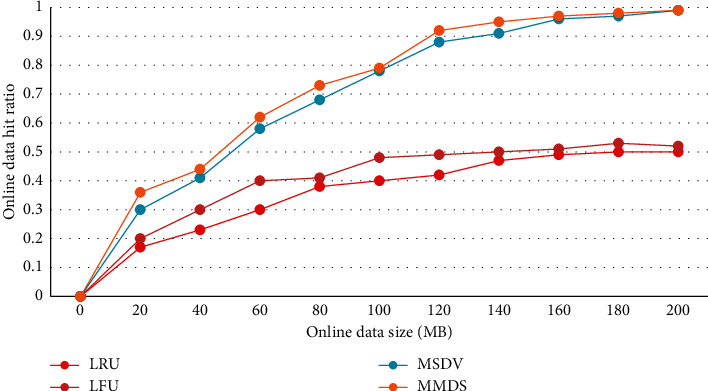
Changes in online data hit rate with online data size.

In order to prove that the migration model is more accurate, classic data migration algorithms (such as LRU, LFU, and MSDV) are used to compare the migration model experiments proposed in this paper.  Figure 3 shows the impact of the amount of online data on the data access rate. As can be seen from the data results in Figure 3, with the increase in online data volume, the data access rate of several algorithms has been improved by different magnitudes. The algorithm in this paper has more advantages in lifting speed and stability, so it has some advantages over other existing algorithms.

**Figure 4:**
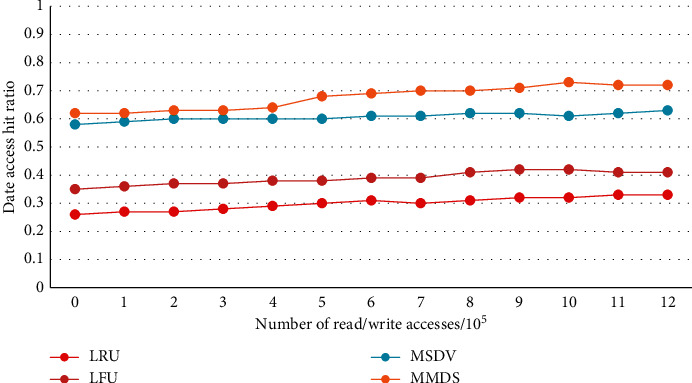
The online data hit rate varies with the number of visits.

Figure 4 shows the relationship between data and access times. In addition to the algorithm in this paper, several existing algorithms are also listed for comparison. It can be concluded from Figure 3 that the access rate of the four algorithms shows a constant upward trend with the increase in the number of read and write I/Os.

### 3.2. Data Mining Algorithm

#### 3.2.1. Classification Analysis

Classification analysis is to understand the public attributes of the same group of objects in the database and create a classification-related model from this. The premise of this step is to analyze and input reasonable training data samples. The sample is composed of a single group or multiple groups of record elements in the database, and each element contains a certain number of field values. These fields are recorded equally. In addition, all records in the sample are marked with a specific identification mark. The goal of classification is to analyze data categories and use specific and appropriate data models to establish a corresponding expression for each category.

#### 3.2.2. Cluster Analysis

Cluster analysis is different from classification analysis. It classifies a group of physical objects or abstract objects (unclassified records) into several categories based on similarity, also known as “non-directed classification.” The distance between them should be as small as possible, and the distance between objects of different categories should be as large as possible. For large cubes, data points are usually not evenly distributed throughout the data space. The data grouping method will detect locations with low population density and then discover the entire distribution model of the data set. The cluster analysis method is suitable for analyzing data that does not have descriptive information or cannot be organized by any classification method and can automatically obtain classification results. The methods used in cluster analysis usually include statistical methods, machine learning methods, and neural network methods.

#### 3.2.3. Association Analysis

Association analysis is the application of association rules in data extraction. This is a data extraction method that has been studied and widely used in recent years. The concept of association rules was proposed by Agrawal, Imielinske, and Swami. This is a simple but practical rule, covered in a descriptive way. There are many development algorithms for association rules, which are subject to unmanaged learning methods, such as APRIORI, sampling algorithm, DIC algorithm, and so on. Association analysis is to investigate the relevance hidden in the data, especially for transaction databases, such as sales data (basket date), to find a form of “90% of customers buying product A while buying product *B*” knowledge.

The information sent by the source includes a limited number of equal exclusive events and complete events, all of which have specific probabilities, expressed in mathematical formulas as follows:

(14) $$\begin{array}{c} H\left(X\right)=-\overset{r}{\underset{i=1}{\sum }}p\left(Xi\right)I\left(Xi\right)=-\overset{r}{\underset{i=1}{\sum }}p\left(Xi\right)\log\;p\left(Xi\right)\text{.} \end{array}$$

Shannon proposed and developed information theory and mathematical information research. Through the uncertainty of various source symbols for measuring the amount of information after the exchange, he proposed a series of concepts:

(1) Self-information. If *X*_1_,…, *X*_*i*_ are the signals sent to the source, before receiving *X*_*i*_, the uncertainty of the receiver of the signal sent to the source is defined as the self-information number *I* (*X*_*i*_) in the symbol.
(2) Entropy of information. The amount of information itself can only reflect the uncertainty of the symbol, and the entropy of the information can be used to measure the total uncertainty in the *X* source, which is defined as follows:
  (15) $$\begin{array}{c} H\left(X\right)=-\overset{n}{\underset{i=1}{\sum }}P\left(Xi\right)\log\;P\left(Xi\right)i\in \left.\left[1,n\right.\right)\text{.} \end{array}$$
(3) Conditional entropy. If the source *X* and the random variable *Y* are not independent of each other, the receiver will receive the message *Y*. Then use the entropy condition *H* (*X*/*Y*) to measure the receiver's uncertainty after receiving the random variable *X*. Receive random variable *Y* and make *X*.
Corresponding to the origin symbol *Yj*, then:
  (16) $$\begin{array}{c} H\left(\frac{X}{Y}\right)=-\overset{n}{\underset{i=1}{\sum }}\overset{m}{\underset{j=1}{\sum }}P\left(\frac{Xi}{Yj}\right)\log\;p\left(\frac{Xi}{Yj}\right)i\in \left[1,n\right],j\in \left[1,m\right]\text{.} \end{array}$$
(4) The average amount of mutual information. Use it to represent the amount of information about *X* that the *Y* signal can provide, expressed by *I* (*X*, *Y*):
  (17) $$\begin{array}{c} I\left(X,Y\right)=H\left(X\right)-H\left(\frac{X}{Y}\right)\text{.} \end{array}$$

Then, introduce other improved ID3 algorithms:

(1) Estimation method based on the information report
In the ID3 algorithm, only changes to the sample classification process are considered, but sometimes, in order to determine attribute *A* value of an instance, it is necessary to perform experimental calculations and so on, and this requires a specific cost. For example, attribute *A* takes values *A*_1_, *A*_2_, *A*_*k*_, *N*_*k*_ and the number of instances of *N*_1_ + *N*_2_ + … + *N*_*k*_ = *N*, where *N* is the total number of training times. Quinlan uses the entropy value *V* (*X*, *A*) of attribute *A* to determine the price to be paid to obtain an example value of attribute *A*.
  (18) $$\begin{array}{c} V\left(X,A\right)=-\overset{k}{\underset{i=1}{\sum }}\frac{Ni}{N}\log\frac{Ni}{N}\text{.} \end{array}$$
When attribute *A* provides the same amount of information *I* (*X*, *A*), the lower the value *V* (*X*, *A*), indicating that to obtain the value of attribute *A*, the cost to be paid is lower. Quinlan defines another selection criterion for testing as follows:
  (19) $$\begin{array}{c} E\left(X,A\right)=\frac{I\left(X,A\right)}{V\left(X,A\right)}\text{.} \end{array}$$
(2) Valuation based on classified information
Cendrowska selected test attributes based on the number of useful information provided by the attributes. The training examples are divided into categories *C*_1_, *C*_2_,…, *C*_*k*_. Let the value of attribute *A* be *A*_1_, *A*_2_,…, *A*_*k*_. Take all |*C*_1_| ≠ 0, set *m*, and define
  (20) $$\begin{array}{c} F\left(X,A\right)=\frac{1}{mk}\overset{m}{\underset{i=1}{\sum }}\overset{k}{\underset{j=1}{\sum }}\log\frac{P\left(\left.Ci\right|Aj\right)}{P\left(Ci\right)}\text{.} \end{array}$$
It is considered that attribute *A* that maximizes *F* (*X*, *A*) should be selected as the test attribute.
(3) Method of estimating by dividing distance
DeMantaras recommends using the distance split method to select test attributes. For example, the classified attributes belong to different categories *C*_1_, *C*_2_,…, *C*_*k*_. If there is a way to divide *X* into indexes *X*_1_, *X*_2_,…, *X*_l_ at a given time, then each instance of *X*_*i*_ belongs to the class *C*_*i*_, which is the most ideal state and is called perfect division. On the other hand, if *X* is divided into subsets based on the possible values of a certain attribute, then *X* is also a class division. If we can define the distance between two divisions, we can choose the one that is closest to the ideal division among the divisions defined by each attribute and define this attribute as the current test attribute. The distance defined by DeMantaras is also based on information and entropy.
*H* (*X*, *C*) defined by this formula is abbreviated as *H* (*C*).
Assuming that *M* = {*M*_1,_*M*_2_,…, *M*_*t*_} represents a specific division of *X*, the entropy of its information is
  (21) $$\begin{array}{c} H\left(M\right)=-\overset{s}{\underset{i=1}{\sum }}P\left(Mi\right)\log\;P\left(Mi\right)\text{.} \end{array}$$
Let *N* = {*N*_1_, *N*_2_, *N*_*t*_} be another division, then the corresponding information entropy is
  (22) $$\begin{array}{c} H\left(N\right)=-\overset{n}{\underset{i=1}{\sum }}P\left(Ni\right)\log\;P\left(Ni\right)\text{.} \end{array}$$
Then the conditional entropy of dividing *M* to dividing *N* is
  (23) $$\begin{array}{c} H\left(\frac{N}{M}\right)=-\overset{t}{\underset{i=1}{\sum }}\overset{s}{\underset{j=1}{\sum }}P\left(MiNj\right)\log\left(\frac{P\left(MiNj\right)}{P\left(Mi\right)}\right)\text{,} \end{array}$$
where *P* (M:N) is the probability that an instance belongs to the *M*_*i*_ class in the division *M* and belongs to the *N* class in the division *N*. DeMantaras defines the distance between two divisions as follows:
  (24) $$\begin{array}{c} d\left(M,N\right)=H\left(\frac{N}{M}\right)+H\left(\frac{M}{N}\right)\text{.} \end{array}$$
DeMantaras uses normal form distance as a metric for selecting test attributes. Normal form distance is defined as follows:
  (25) $$\begin{array}{c} D\left(M,N\right)=\frac{d\left(M,N\right)}{H\left(MN\right)}\text{,} \end{array}$$
where
  (26) $$\begin{array}{c} H\left(MN\right)=-\overset{l}{\underset{i=1}{\sum }}\overset{s}{\underset{j=1}{\sum }}P\left(MiNj\right)\log\;P\left(MiNj\right)\text{.} \end{array}$$
If attribute *a* refers to the division method of *L*, then because the division method of *C* = {*C*_1_, *C*_2_,…, *C*_*i*_} is an ideal division, it is also a division method. DeMantaras takes *D* (*L*, *C*) as the criterion for selecting test attributes. A simple derivation shows that
  (27) $$\begin{array}{c} \frac{H\left(C\right)-I\left(X,a\right)}{H\left(LC\right)}=1-D\left(L,C\right)\text{.} \end{array}$$
(4) Optimization algorithm
The MID3 algorithm is an improved solution to the problem of ID3's weak ability to learn simple logical expressions. Let *A* be a candidate attribute; *A* has *v* attributes, and the corresponding probabilities are, respectively, *P*_1_ … *P*_*V*_, which conforms to the principle of minimum entropy (*B*, *B*_2_,…, *B*) for the extended information of attribute *A*. If, in the *v*-node attribute, the corresponding information entropy is *E* (*B*_1_), *E* (*B*_2_),…, *E* (*B*_*V*_), then
  (28) $$\begin{array}{c} E^{\prime }\left(A\right)=\overset{v}{\underset{i=1}{\sum }}{Pi}^{*}E\left(Bi\right)\text{.} \end{array}$$

## 4. Design and Practical Application of National Fitness Sports Movement Analysis System

### 4.1. Motion Capture Analysis of Sports Competitions

Bodenheimer and Fontaine successively designed the calculation of the total inertial motion of the body from the original data to the final posture capture. Preliminary research first focused on the software level, assuming the human skull as an articulated steel structure, using offline optimization methods to determine the bone length, and using reverse chains to perform the movement in kinematics. However, this paper cannot solve the problem of multiparameter schemes well, and the accuracy of restoration is limited. The latter is committed to building motion capture devices in a new generation of human-computer interaction interfaces, recovering and calculating raw data more from the hardware level, taking into account the selection and wear of sensors, and the entire process involving calculations and explaining the errors in the process.

The measured error behavior includes the measurement error of the original sensor and the unnecessary integral drift from the original data to the behavior calculation process. The basis for improving the quality is high-quality components and higher prices. For the latter, the integral inertial drift is performed through two integral processes from motion acceleration to displacement and a causal process from angular velocity motion to characteristics. As an inevitable problem of capturing inertial motion, you can refer to the world provided by the magnetometer. However, in this respect, in addition to the measurement error of the magnetometer itself, there is also the risk of instability around the magnetic field and the surrounding magnetic damage to the ground, including hardware, and the magnetic measurement of each sensor is also very different.

Changes in soft tissues (such as muscles during exercise) directly lead to errors in the binding relationship between the inertial sensors and the correct positions of the limbs. This is also the most difficult problem in personality capture during exercise. Today, the most common muscle modeling method is the SMPL model proposed by German scholars. The model takes *β* and *θ* as input, representing 10 parameters related to the shape of the human body, and 75 parameters related to the posture of the human body can simulate muscle depression and muscle swelling during exercise. The method used by Taetz et al. is more flexible. He regards the truncated cone as each branch of the human body. By inputting the radius of the two ends of the foot, the radius of any position of the foot can be calculated so that the deviation between the spatial position of the sensor and the central part of the foot can be used as a variable. If you replace the cone cut in this way with another geometric pattern, the shape of the limb will be better simulated.

The contact between the human body and the external environment provides motion constraints to capture human motion, which can correct the position of human bones and reduce the impact of overall inertial drift. Among them, the contact between the foot and the ground is the most common, and it is also the most studied topic. There are three algorithms for determining plantar contact: The first method captures the speed or intensity of the foot contact response during exercise and then sets the contact threshold for evaluation. If the acceleration/velocity is less than the threshold or if the contact reaction force is greater than the threshold, the person is considered to be in contact with the ground. However, this method is only suitable for regular exercise variants, so the decision to do a quick exercise or other unconventional exercise is not ideal. The second method is to calculate the probability of back contact. After comprehensively analyzing the performance of influencing factors such as acceleration and reaction force that affect different actions, the best experimental results are obtained. In addition to the above methods, some researchers have also tried to use neural network methods, but this method requires sufficient training data to support an accurate model.

Non-functional fitness system requirements include server response speed, system security, data integrity, and so on. The specific analysis is as follows: the fitness system is constructed through a model that separates the front and back ends, and several rear modules are connected to the storage management platform of the system. The response time from the server to the client should not exceed 2 seconds. Because the user is allowed to be online at the same time and the deployment environment after the online distribution is distributed, the server must record the user's login information and does not allow the user to access other users' data information. For non-character fields in the system, when retrieving or calculating values, the accuracy of the value must be required. For example, for fields related to money, the system needs to keep two decimal places. Except for special external requests, other data variables retain four digits after the decimal point. The fitness system uses IDEA as a development tool amd HBase as the best business data storage system; all modules are built in the layer, and Spring Cloud Feign is used for remote calls. In the cluster solution, after the user logs in, if he accesses other modules, the user will not be allowed to log in again, that is, the combined identity authentication of the user must be ensured.

### 4.2. System Architecture Design

Each module of the national fitness system software service architecture based on HBase storage and microservice architecture is interdependent and relatively independent, and each module is interdependent, and each module provides relatively independent services.

If the fitness system is divided into a small module, such as user module, venue module, course module, and so on, and weighted interfaces in each module, the service registry center is born, which helps the fitness system manage and monitor each module call. In a distributed system, the interaction between services is very complicated. Certain services will inevitably fail, causing remote call lines to block other services that depend on them, and ultimately destroy the entire service system.

Each module or project has many configuration files, which means that the system can dynamically adapt to different environments. In the early days of a standalone system, it was easy to change the configuration file on this node. So all you have to do is to find the configuration file, edit it, and restart the service. However, for hundreds or thousands of microservice machines, it is often unreasonable to change the maintenance. In order to change the configuration files faster and smarter, the system must use the service configuration center to manage the system configuration files in an integrated manner.

The microservice architecture is a distributed architecture. Service units are divided according to activities. Microservice systems usually consist of several small service units. Due to a large number of service units, the complexity of the system is very high. If there are errors or exceptions in a particular service, it will be difficult to find problems and find service exceptions. In order to solve this problem, the fitness system must track the provided links and monitor the call sequence and demand flow of each request, so as to achieve a clear understanding of the path of each request in each service to achieve the purpose of the service.

### 4.3. Business Process Design

**Figure 5:**
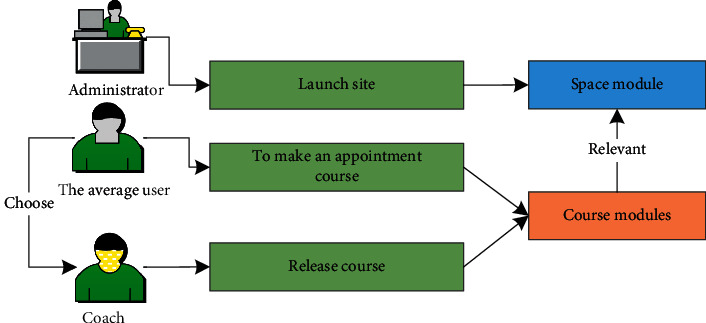
The overall structure of the fitness system.

As shown in Figure 5, this is a diagram of the relationship between different modules in the fitness system. The venue manager assigns location module contacts by publishing and canceling locations. Ordinary users contact the venue module and coaches by booking venues, canceling venues, and selecting fitness coaches. Coaches can post fitness courses, so they can establish connections by posting courses and course modules. Ordinary users can order fitness courses to contact course modules by booking courses.

Booking a course means that ordinary users can book the fitness course they want, and the system will send the user a reminder of the course. For the planned courses, users can filter by course name, course time, course address, and other filtering conditions to choose the course that suits them.

The booking planning process is as follows:

1. Course inquiry. After entering the course reservation page, the user enters the filter conditions: course name, course time, course address, and other data and then initiates a system request. The system first checks the token information and authorization information and guides the course module after configuring the rules through the gateway. The course module will transmit parameters, then check the parameters, and finally query the data in the database, and the caller at the front desk will return the result.
2. Book a course. After the user selects a course, click the selected course to send a background request. Similarly, check the token at the gateway. After correct verification, it will pass the course module and finally store it in the database after checking the company's course parameters, and the operation will quickly return to the former caller.
3. Remind users to notify. If the user has successfully booked the course, the course module will send a message to the message module to inform that the course has been successfully booked. The message module saves the retained course data in the pre-published course version. After using the synchronization thread, it periodically requests a primary message. If the data is found, it sends a course notification message to the students who registered for the course and then deletes the entry from the pre-post.

The process of designing and distributing the site is as follows:

1. Venue inquiry. After entering the reservation page of the venue, the user enters the filter conditions: location name, location address, and other data, and then initiates a system request. The system first checks the token information and authorization information and then routes the gateway through the gateway configuration rules; the positioning module analyzes the parameters, then checks the parameters, and finally queries the data in the database and returns the results to the front-end caller of the gateway.
2. Site reservation. After the user selects a location, click Book to submit a background request. Similarly, check the token at the gateway. After the verification is successful, it is sent to the location module, and the company's location parameters are checked and finally stored in the database, and the quick success of the operation is returned to the previous caller.
3. Reminding users to use the venue. If the user has successfully booked the venue, the location module will send a message to the message module to inform that the reserved location has been successfully booked. The message module will save the data stored in the location area before starting and then use the synchronization thread every minute. Ask for the pre-registration once. If data is found, a location notification message will be sent to the user, and the pre-registration record will be deleted after publishing.

Course management means that the coach completes the course information in the system and then releases it. If a student subscribes to this course, the system will send a notification message to the teacher of the course. Coaches can analyze the number of students who have booked courses and information about students. At the beginning of the course, the system will send a reminder to the coach. Coaches can also cancel courses that have not yet started, and the system will send a notification of course cancellation to individual coaches and students.

The course management process is as follows:

1. Publish courses. After the instructor is connected to the system, he will enter the course start page. The instructor clicks the Add button and then enters the course information, such as the course name, start time, and other information. Then click Publish; the client sends a background request; and the background confirms the authorization symbol and information. After the verification is successful, the gateway will pass the course module, and then the course module checks the parameters, saves the data in the command table, and sends a message that it has been successfully started. Finally, the message that the course has been successfully published is returned to the front end.
2. Release the course. The coach is informed that the course has been successfully released. The message module uses the time thread to request the pre-site to publish a course every minute. If there is data in the query table, a message will be sent announcing the successful release of the coach, and then the record will be deleted from the pre-release.
3. Cancel the course. After connecting to the system, the coach enters the published course page; the coach selects the published course and then chooses to cancel the course. The client sends a request to cancel the background process, and the background confirms the token and authorization information. After the verification is successful, the gateway will guide the course module, and then the course module will verify the parameters and query the database to overwrite the default signaling settings. Continue to send the message module; the message module will notify all students and coaches of the cancellation of the course; and finally, the course cancellation message will be successfully returned to the front end.

### 4.4. Data Sheet Design

All informational data, such as ordinary users, courses, venues, and various related data, have been successfully uploaded to the NoSQL database. Due to the massive amount of system data, the HBase database is used in specific implementations. HBase is a key-value storage library, which is a NoSQL database oriented to column families. The efficiency level of the key query is in milliseconds. Analyze the aforementioned system data relationship model, combined with the requirements of the upper-level application and the time requirements of millisecond-level queries that do not have the same node attributes and known relationships. By designing the business process, multiple tables are established for the fitness system, and a single question is based on the secondary index of the secondary question.

The user node integrates ordinary users, coaches, and venue managers and mainly stores basic user information. There are several ways to access username-based access, so use user ID as the row key. The table is divided into two columns: userinfo and count. In the userinfo column, appropriate personal information is stored. Due to the scalability of the HBase model, all attribute fields that can be added in the future can be added directly. This information is not always updated and read at the same time, so putting them in the same column series can reduce the I/O read and write latency. The number in the column is used to store information in bulk, such as the number of selected courses, the number of reserved venues, and the number of open courses. If data is entered in the user table, the corresponding value is automatically incremented using the HBase incremental extension. In this way, you can directly query through the primary key to avoid the main defect of the entire scan of the statistical query table in the HBase database.  Table 1 lists the node table used.

The course table stores the basic information of course nodes, which is also divided into two series. The Classinfo series stores information about the course name, start time, end time, coach, and other corresponding information that is not frequently changed. The course node table is shown in Table 2.

The site table stores basic information about site nodes. It is also divided into two column families. In the areainfo series, information about venue names, venue addresses, venue publishers, and other related information are stored. The site node table is shown in Table 3.

The venue user relationship table stores the venue reserved by the user and the site information released by the venue administrator. The table is divided into two columns: info and time. The info column stores the user ID, venue ID, user venue relationship, and other information; the time column stores the time when the venue is open to the outside world or the time scheduled by the user.  Table 4 shows the relationship between users and scheduled venues.

## 5. Conclusion

This article uses the DARPAID3 evaluation data set developed by MIT Lincoln Laboratory for experimental testing. In the first week of recording, we randomly selected 9,267 audit records as the original training data and obtained the judgment table based on the above-mentioned data processing method. The table has 40 attribute states, which are evaluation attributes. Randomly select 10,985 data from the second week as the test data. This article selects HBase storage technology and microservice structure for the fitness system and develops a national fitness system based on HBase and microservices. The system uses HBase to store health information, scheduled course information, coaching information, and venue information about the nation's fitness people and uses a microservice architecture to divide the system into user modules, regional modules, and course modules, which reduces system latency, and the scalability of the system is improved, and a system is created for the services of ordinary users, fitness coaches, and webmasters. This system is essential for the rational use of fitness facilities and the improvement of national health.

## Figures and Tables

**Table 1:** User node table.

RowKey	Family	Column	Description
<UserId>	Cfl: userinfo	UserName	Username
		PhoneNum	Phone number
		Sex	Gender
		Email	Mailbox
		Pwd	Password
		Remark	Remarks
		Flag	Logout flag
		RoleName	Character
	C12: count	ClassCount	Number of courses
		OrderCount	Number of venues ordered
		ClazzCount	Number of courses
		GroundCount	Number of release venues

**Table 2:** Course node.

RowKey	Family	Column	Description
<ClassId>	Cfl: classinfo	Userid	Teaching coach
		ClassName	Course name
		StartTime	Starting time
		End time	End time
		Desc	Description
		Flag	Delete course logo
	Cf2: count	orderCount	Number of students in class

**Table 3:** Site nodes.

RowKey	Family	Column	Description
<Area Id>	Cfl: areainfo	Userid	Venue publisher
		Area Name	Venue name
		Address	Venue address
		Flag	Post status
		Latitude	Site dimension
		Longitude	Site longitude
	Cf2: count	Order count	Number of times booked
		PushCount	Number of releases

**Table 4:** User site relationship table.

RowKey	Family	Column	Description
Hashkid>	Cfl: info	Userid	User ID
		Areald	Venue ID
		Relation	User site relationship
		Flag	Status
	Cf2: time	StartTime	Scheduled start time
		EndTime	Scheduled end time

## Data Availability

The data used to support the findings of this study are available from the corresponding author upon request.
